# The Role of Equine Herpesvirus Type 4 Glycoprotein K in Virus Replication

**DOI:** 10.3390/v4081258

**Published:** 2012-08-07

**Authors:** Walid Azab, Abuelyazeed El-Sheikh

**Affiliations:** Department of Virology, Faculty of Veterinary Medicine, Zagazig University, Egypt; Email: eaa000@yahoo.com

**Keywords:** EHV-4, BAC, gK

## Abstract

Equine herpesvirus 4 (EHV-4) is an important equine pathogen that causes respiratory tract disease among horses worldwide. Glycoprotein K (gK) homologues have been identified in several alphaherpesviruses as a major player in virus entry, replication, and spread. In the present study, EHV-4 gK-deletion mutant has been generated by using bacterial artificial chromosome technology and Red mutagenesis to investigate the role of gK in EHV-4 replication. Our findings reported here show that gK is essential for virus replication *in vitro* and that the gK-negative strain was not able to be reconstituted in equine cells. It is noteworthy that these findings agree with the previously published study describing gK deletion in other alphaherpesviruses.

Equine herpesvirus 4 (EHV-4) is a member of the *Alphaherpesvirinae* subfamily, genus *Varicellovirus* [1,2]. The virus is endemic in horse populations throughout the world and it causes infection that usually remains restricted to the upper respiratory tract [3]. Previous studies with herpes simplex virus type 1 (HSV-1), pseudorabies virus (PrV), duck enteritis virus (DEV), and EHV-1, the close relative of EHV-4, have shown that gK plays a major role in virus entry and replication as well as it is required for efficient cell-to-cell spread and virus egress [4,5,6,7,8,9]. With the molecular tools now at hand, we were able to investigate several EHV-4 genes during the last few years [10,11,12,13,14]. While the role of gK has been established for some herpesviruses, no data are currently available about EHV-4 gK and its role in virus replication.

In this study, we reported the construction and characterization of the virulent EHV-4 strain TH20p following the deletion of the gK gene. We could delete gK precisely from the EHV-4 genome backbone. 

EHV-4 BAC clone pYO03 [11] (Figure 1a) was maintainedin *Escherichia coli* (*E. coli*) EL250 strain (a kind gift from Dr. Neal G. Copeland). Deletion of gK was done via two-step Red recombination as previously described [15]. Briefly, PCR primers, gKkanF cctgggtgtcaggatttttatagagacttacaagccgcgcccactagttaaggatgacgacgataagtaggg and gKkanR atgtgcaccatttttacgctagaggtgaacagagcaaaataatatacacacaaccaattaaccaattctgattag, were designed to have recombination arms of 60 nucleotides that enabled the substitution of the gK gene by the kanamycin-resistant (*Kan^R^*) gene amplified out of plasmid pEPKan-S, a kind gift from Dr. N. Osterrieder [15]. PCR products were digested with *Dpn*I in order to remove residual template DNA. Transfer fragments were then electroporated into EL250 containing EHV-4 BAC. Kanamycin-resistant colonies were purified and screened by PCR and restriction fragment length polymorphism (RFLP) to detect *E. coli* harboring mutant clones. PCR analysis revealed that primers, gKF aagttttaatcagtaggtgt and gKR gcaacaataaaatgtgcacc, binding to the outside of the deleted part of gK yielded a PCR product of around 1000 bp in case of parental EHV-4 and EHV-4∆gK, as gK and *Kan^R^* gene both are having around 1 kbp in length, (Figure 1b, left panel). While using one sense primer that binds to *Kan^R^* gene and another anti-sense primer that binds to the outside of gK yielded a PCR product of 1000 bp in case of the mutant EHV-4∆gK only (Figure 1b, right panel)**. **gK is located within a 5.9 and 2.7 kbp *Eco*RI fragments, due to the presence of an *Eco*RI site. The bands of these two fragments were disappeared and replaced by a single band of 8.6 kbp in size in the case of EHV-4ΔgK due to the presence of the *kan^R ^*gene, which has no *Eco*RI sites (Figure 1c). These results and those obtained by DNA sequencing (data not shown) confirmed the correct and exact insertion of the *Kan^R^* gene instead of the gK gene.

**Figure 1:**
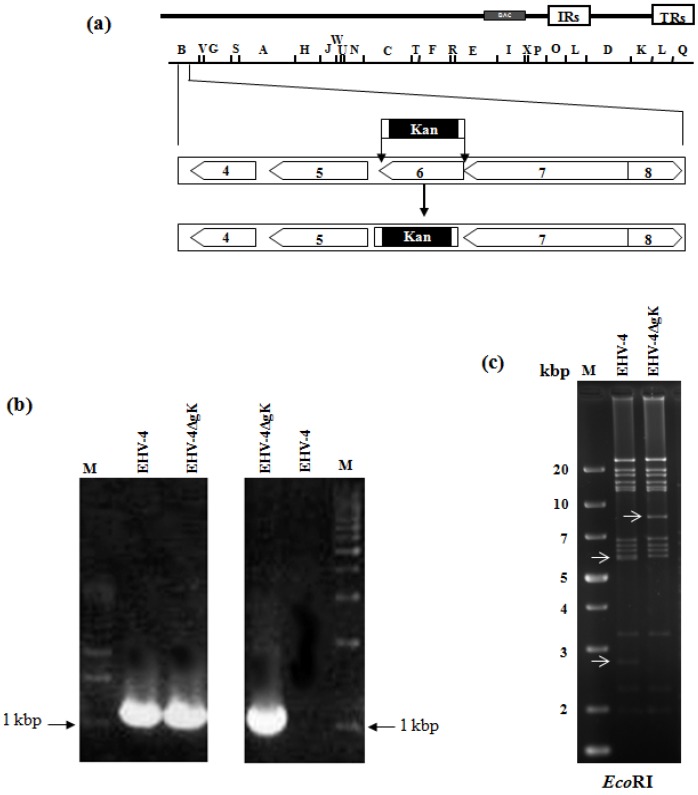
**Mutagenesis and generation of gK deleted mutant.** (a) Schematic diagram of the procedures used to delete the gK gene from EHV-4 BAC. Schematic representation of the genomic organization and the *Bam*HI restriction map of EHV-4 BAC pYO03 [11] is given. The genomic organization of the domain that encodes the genes from 4 to 8 [16] is depicted within the *Bam*HI B restriction fragment. The PCR cassette conferring *Kan^R^* gene was inserted into the gK locus of pYO03 using Red recombination. (b,c) Identification of EHV-4∆gK by a combined PCR/RFLP analysis. PCR products from parental EHV-4 and mutant virus were electrophoresed in a 1 % agarose gel. Primers binding to the outside of the deleted gK (left panel) as well as one sense primer that binds to the *Kan^R^* gene and another anti-sense primer that binds to the outside of gK (right panel) were used. A molecular weight marker (lane M) was included (b). Purified DNAs from EHV-4 and EHV-4ΔgK were digested with *Eco*RI (c). Fragments in the mutants that appeared or disappeared as a consequence of the insertion of *Kan^R^* gene instead of gK are marked by arrows.

To determine whether the gK gene is essential for EHV-4 replication in cell culture, parental EHV-4 and EHV-4ΔgK DNA were purified using large construction kit (Qiagene) and transfected into HEK293 cells using Lipofectamine 2000 (Invitogen) (Figure 2a and b). Three days later, the supernatant and cells were collected and used to infect confluent NBL-6 cells. Our results showed that while the parental EHV-4 was able to grow and produce green plaques on NBL-6 cells, EHV-4∆gK was not able to grow in these cells over time. Only single cells were infected and there was no development of plaques, indicating that gK is essential for virus replication *in vitro* (Figure 2c and d). For generating NBL-6 cells that express EHV-4 gK (NBL-6/gK), NBL-6 cells were transfected with the recombinant pcDNA_gK or pcDNA3_GFP (control) plasmids using electroporation (260 V, 1050 μF and 335 Ω) as described before [17]. However, trials to complement gK functions by a cell line that express the authentic gK failed. A possible explanation may be that the protein is not expressed in all transfected cells due to the very low transfection efficiency of NBL-6 cells. Another possible explanation may be that expressing gK under the control of the HCMV IE promoter found in pCDNA3.1 plasmid may result in different expression levels and/or timing, which are important for gK function [18,19]. These results seem to be comparable to those in case of EHV-1, HSV-1, and PrV where incomplete complementation of the absence of gK-encoding sequences in recombinant viruses was seen [6,20,21]. 

**Figure 2:**
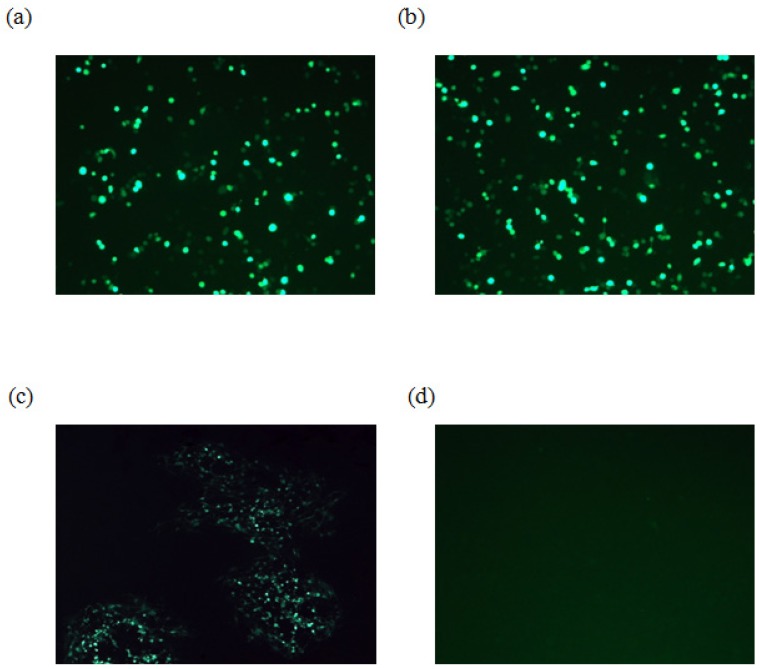
**Infection of NBL-6 cells with EHV-4ΔgK. **HEK293 cells were transfected with either EHV-4 parental DNA (a) or EHV-4ΔgK DNA (b). Viruses were collected from infected HEK293 cells and used to infect naïve NBL-6 cells. (c) Cells infected with parental virus. (d) Cells infected with EHV-4ΔgKvirus. Transfected and infected cells appear green as all viruses express EGFP. Cells were inspected with a fluorescent microscope (Zeiss) and images were taken with a CCD camera (Zeiss).

In summary, our data showed that EHV-4 gK is essential for virus replication *in vitro*. However, the exact role of gK in virus life cycle is still to be elucidated. 
